# Notes and News

**Published:** 1892-03-19

**Authors:** 


### Notes and News.

COLLECTED AND COMMUNICATED.

In respect to the enlargement of the paper, we have to state that the institutional portion will be opened on April 2nd by a series of papers, by Mr. Burford Rawlings, on "Hospital Indigence and its Cure."

At a meeting, on the 9th inst., of the Committee of the New General Hospital, Birmingham, the three prizes for the best competitive designs were awarded to Mr. Henman, of Birmingham; Messrs. Worthington and Elgood, of Manchester; and Mr. F. B. Osborne, of Birmingham. Mr. Henman's designs were unanimously adopted, and resolutions were passed authorising estimates to be obtained for carrying them into effect. Mr. J. C. Holder, who presided, made an appeal for further contributions in order to erect a building worthy of the town, and by way of impetus, himself promised an additional £1,000. It was originally estimated that the building would cost £80,000, but it is now understood this sum will be much exceeded.

To readers of The Hospital familiar for the most part with the sameness (dare we say?) of hospital accounts, the following list of charges found in an old record in Winchester Cathedral, and dated A.D. 1182, may show that the matter-of-fact may sometimes be a little startling. The account runs thus:—

| To work done— | s. | d. |
|---|---|---|
| To solderynge and repairynge St. Joseph ... ... | 0 | 6 |
| ,, clengyne and ornamentynge ye Holy Ghost ... | 0 | 6 |
| ,, reparynge ye Virgyne Mary before and behind, and makyne a new child .. ... ... ... ... | 4 | 8 |
| ,, screwing a nose on the Devil, puttynge a horne on his hede, gluynge a byt on his tayle ... ... | 5 | 6 |
| | 11 | 2 |

The influenza epidemics of the last two years present distinct and interesting features. In the first epidemic the after effects though marked by great nervous depression in innumerable cases were followed by few if any cases of marked mental disorder. There were, however, numerous cases of hysteria in both sexes which gave serious trouble at the time, but the patients invariably made good recovery. In the last epidemic which is now passing away, mental disorders have been a marked feature. During the present year the demand in consequence for mental nurses has been unprecedented; this is due to the large number of cases of insanity caused by the influenza. The insanity, which was first of one type, religious mania and hallucinations, has since assumed many forms. The first form is attended by great danger to the patient and requires prompt treatment. It is too early to determine how far the effects will be permanent, but where the cases have been recognised at the outset, there is every reason to hope that no permanent ill effects will result. We have thought it desirable to call attention to these special features of the epidemic so as to allay undue alarm and to aid the friends of such patients by urging upon them the importance of prompt measures in the directions indicated.

The Belgian Gynæcological and Obstetrical Society intend starting an International Congress of Obstetrics and Gynæcology, to be repeated at intervals of four years, alternately in Belgium and Switzerland. The first Congress is to be located at Brussels, and will be held from the 14th to the 19th of September in this year. The three subjects chosen for discussion on this occasion will be opened by Dr. Seqond, of Paris; Dr. A. Martin, of Berlin; and Dr. Berry Hart, of Edinburgh. Subscriptions for members on the foundation is 300 francs (which covers all subsequent charges), and for members taking part in the first Congress 30 francs, which entitles them to reports of the proceedings of all succeeding meetings. There will be an international exhibition of gynæcological and obstetrical instruments and apparatus in connection with the first Congress, which will take place on the premises of the Congress and at the Brussels Maternity Hospital. All enquiries should be addressed to the Secretary-in-Chief, Dr. Jacobs, 12, Rue des Petits-Carmes, Brussels. Communications should be sent before July 1st.

The 75th annual meeting of the Royal Hospital for Women and Children, was held last week at the Mansion House, the Lord Mayor presiding. The report stated that the year had been a very trying one for all hospitals, but the Committee thought it satisfactory that the income had been fairly well maintained. The expenditure for keeping open the whole of the wards, however, was not nearly met, and the deficit of £600 with which the year was started had increased to £1,500. The work of the institution had been considerably extended, the number of in-patients being 586, against 509 in 1890; out-patients, 6,863 against, 6,710; attendances, 27,988, against 27,154; and dental cases, 263, as against 310. The Committee concluded by making an urgent appeal for help, and pointed out that the ordinary income was about £2,000 per annum less than the sum needed. The Lord Mayor made a speech, which will, it may be hoped, excite a large amount of sympathy, and be the means of inducing those who are able and willing to assist in any benevolent work, in which they are assured that their money will be well spent, to send in contributions that will enable the managers of one of the best and most economically worked charities in the metropolis to utilise the building and its resources to the fullest extent.

The unfortunate accident through the overturning of a lamp at the St. Pancras Club, during a children's entertainment, is only one instance out of a large number of catastrophes arising from the use of paraffin. Such unfortunate occurrences as that at the St. Pancras Club are not wholly without their uses if we learn as we should, the lessons they convey. Our Board schools are increasing their curriculum with every conceivable subject which may possibly be of use to the scholars in future life. The tendency is on the whole towards practical knowledge, though technical education is more rapid in the same direction. Under the heading of domestic economy, Board schools endeavour to instil into the minds of the pupils a certain amount of common-sense rules for daily use. Surely one of the most valuable lessons that could be learnt by all classes of society would be conduct to be observed in case of fire arising from any cause, but above all in the case of the upsetting of lamps. Paraffin and other mineral oils are fast becoming universal favourites amongst the poorest class of the community on account of their cheapness, and accidents from this source will multiply accordingly. Mrs. Barber's prompt action in supplying some of her clothing to extinguish the flames at St. Pancras, gives an instance of practical common sense, which it would be well if it were more general. A few simple rules of action instilled into the minds of children would bear practical fruits, and we should not have to exercise unavailing sympathy with little sufferers from panic, such as those conveyed to the Royal Free Hospital recently. We have ourselves been present at a conflagration where 200 children and only six or seven grown up people were present, and perfect order prevailed and assistance was effectually rendered by some of the elder children present. It is by no means an impossible task to instil self-control and common sense into children, and the example of the few forms the initiative to the many.

